# Renal function after partial nephrectomy following intra-arterial embolization of renal tumors

**DOI:** 10.1038/s41598-020-78461-5

**Published:** 2020-12-07

**Authors:** Germain Bréhier, Antoine Bouvier, Louis Besnier, Serge Willoteaux, Cosmina Nedelcu, Thibaut Culty, Christophe Aubé, Pierre Bigot

**Affiliations:** 1 Radiology Department, University Hospital, CHU Angers, 4 rue Larrey, 49933 Angers, France; 2 Laboratoire HIFIH, EA 3859, UNIV Angers, 49045 Angers, France; 3 Urology Department, University Hospital, 49933 Angers, France

**Keywords:** Surgical oncology, Urology, Kidney

## Abstract

Laparoscopic Partial Nephrectomy (LPN) after intra-arterial Embolization of renal tumors (LPNE) in a hybrid operating room allows renal tumor enucleation without dissection and clamping of the renal pedicle. The purpose was to assess the potential negative impact of embolization on the renal function. This prospective monocentric study included all patients treated with LPNE between May 2015 and June 2019. Clinical data was collected and incorporated into the UroCCR database (NCT03293563). Glomerular Filtration Rate (GFR) and Computed Tomography Renal Volume (CTRV) were compared before and after 6 months following LPNE. The mean post-operative GFR was 86.6 mL/min (SD 22.9). The mean GFR loss was 9.4% (SD 15.1) and the median renal parenchyma loss was 21 mL (SD 20.6). Using a threshold of 25% GFR loss, age was the only significant predictive factor of renal function impairment according to bivariate (59.5 vs 69.3 years, p = 0.017) and multivariable analysis (OR 1.075, CI 1–1.2], p = 0.05). Significant renal function impairment was not correlated with the renal parenchymal volume loss (OR 0.987, CI [0.95–1.02], p = 0.435). Renal function impairment after LPNE seems to be comparable to other techniques of partial nephrectomy.

## Introduction

As a result of the increasing use of cross-sectional imaging, 40–50% of new renal cell carcinomas are now detected at T1 stage (localized and size < 7 cm) enabling a significant reduction in mortality rates in developed countries despite a worldwide increasing incidence^1^. Partial nephrectomy is now the standard surgical treatment for these tumors, especially when smaller than 4 cm (stage T1a)^2^. Improved knowledge and techniques now allow nephron-sparing surgery, reducing the risk of cardiovascular events and morbi-mortality related to impaired renal function^3,4^.

Feasibility and positive clinical outcomes of a new technique, Laparoscopic Partial Nephrectomy (LPN) after intra-arterial Embolization of renal tumors (LPNE) in a hybrid operating room, have been established in recent studies^5,6^. Most importantly, pre-operative embolization avoids per-procedural hilar clamping commonly used in partial nephrectomy procedures for bleeding control, since hilar clamping time is presumed to be a modifiable surgical risk factor for decreased renal function after partial nephrectomy^7,8^. While the effect of the duration of ischemia on renal function is still a matter of debate, post-operative renal function is clearly correlated with the quality and quantity of the residual renal parenchyma ^7,9^. LPNE allows surgery with zero ischemia but requires intra-arterial injection of an iodinated contrast medium and the sacrifice of a small portion of healthy parenchyma.

The purpose of this study was to assess a potential significant renal function impairment following LPNE and to identify potential predictive factors for significant impaired renal function.

## Materials and methods

### Population

The clinical data reports for the study were collected and incorporated into the UroCCR database (French Research Network for Kidney Cancer, ClinicalTrials.gov Identifier NCT03293563), which is IRB-approved (*Comité Consultatif sur le Traitement de l'Information en Matière de Recherche dans le domaine de la Santé*) and obtained the CNIL (*Commission Nationale de l'Informatique et des Libertés*) authorization number DR-2013-206. All methods were carried out in accordance with relevant guidelines and regulations. This prospective monocentric study included all patients treated for a renal tumor between May 2015 and June 2019 by LPNE.

The inclusion criterion was one single localized kidney tumor. The indication for partial nephrectomy was validated by a multidisciplinary uro-oncologic board.

Patients who underwent Magnetic Resonance Imaging (MRI) instead of Computed Tomography (CT) scans before surgery, patients who did undergo CT scans but not performed in our institution and patients with a single kidney were excluded.

### Procedure

**Figure 1:**
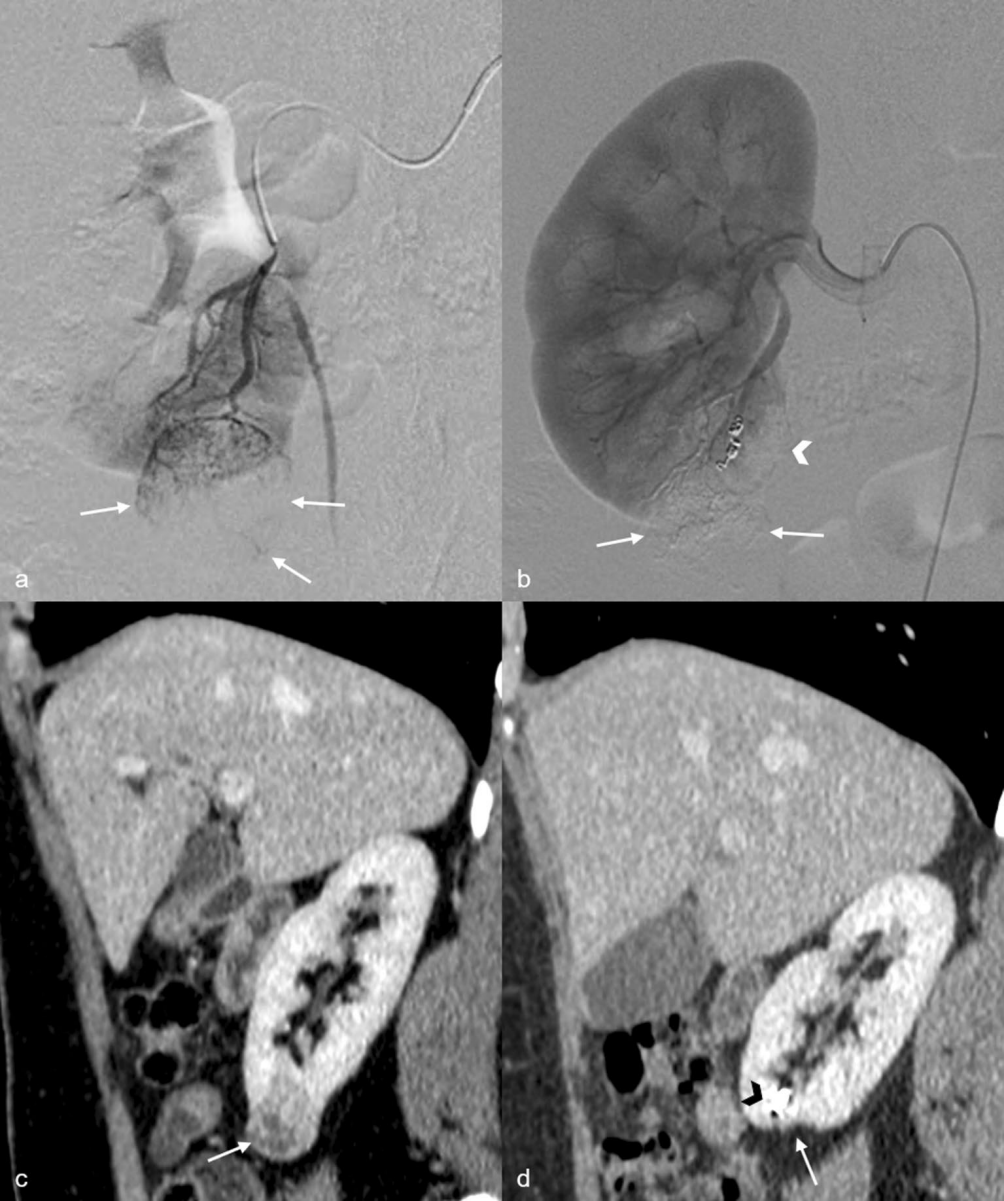
44-year-old woman operated on for right kidney renal clear cell carcinoma (pT1a, complete resection). (**a**) Selective arteriography shows a tumoral blush at the lower pole of right kidney (arrows). (**b**) After embolization, the renal arteriography shows complete tumoral devascularization (arrows) with minimal defect (arrowhead) of the peri-tumoral renal parenchyma. (**c**) Pre-operative CT scan shows the lower polar tumor (arrowhead). (**d**) Sagittal sections of a tubular time CT scan 6 months post-operatively show the operating area with minimal cortical thinning (arrow) and hyperdense embolization material (arrowhead).

All combined procedures were performed under general anaesthesia in a hybrid operating room (Discovery IGS 730, GE Healthcare, Waukesha, WI). Concerning the unfolding of the procedure, first of all, an interventional radiologist performed a super-selective intra-arterial embolization of the renal tumor, then a surgeon carried out the laparoscopic partial nephrectomy. All embolization and surgical procedures were conducted as previously described (Fig. 1)^5^.

### Data and analysis

Pre-operative patient clinical data and tumor characteristics were retrieved: age, sex, BMI, tumor size and renal tumor complexity score (R.E.N.A.L. nephrometry score; score of 4–6: low complexity; score of 7–9: moderate complexity; score of 10–12: high complexity)^10^.

It is well established that the least invasive and most innocuous way to model renal function is the estimation of the glomerular filtration rate (GFR) using standard formulas (according to Cockcroft-Gault or MDRD) mainly based on creatinine blood levels. Furthermore, excellent correlation has been shown between isotopic imaging methods presumed to obtain accurate measurements of glomerular filtration rates and CT measured renal volume (CTRV) used for pre-operative management and oncologic follow-up^11^. CT based volume assessment techniques have already been used to calculate differential kidney function, to show the dominant kidney prior to kidney donation and even to estimate the risk of chronic kidney disease after partial nephrectomy^12–14^. We therefore chose to use GFR (estimated by MDRD formula) and parenchymal renal volume (CTRV) as reflective of renal function.

Creatinine levels and MDRD clearance were collected as the pre-operative CT scans were performed and at 6 months follow-up after surgery. All CT scans were conducted according to a multiphasic protocol including an initial non-contrast-enhanced phase followed by an arterial and/or parenchymal contrast-enhanced phase after intravenous injection of an iodinated contrast medium. All images were transferred to a workstation running personal computer-based software (SYNAPSE 3D, Fujifilm Corporation, Tokyo, Japan) already used in previous studies concerning renal volume calculation^15–17^. Using enhanced CT phases, the aforementioned software first performed an automatic extraction of the volume of both kidneys then adjusted by the operator using different correction tools to obtain the definitive renal volume. All analysis was performed by a junior and a senior radiologist. Ipsilateral and contralateral kidney volumes were measured excluding vessels, urinary tract, tumor volume (in the pre-operative work-up), sequelar unenhanced areas or spontaneously hyperdense material corresponding to embolization devices (in the post-operative work-up).

Procedure-related data were collected: embolization procedure time, number of subsegmental arteries embolized, volume of administrated iodinated contrast media, laparoscopy time, blood loss volume, requirement of hilar clamping or embolization for secondary bleeding.

### Statistical design plan

We compared data before and 6 months after LPNE. For comparison, the × 2 test or Fisher’s test were used for qualitative variables, and Student’s t test was used for quantitative variables. Significant GFR loss was considered when GFR was reduced by 25% after surgery. This threshold was already used in the elaboration of a nomogram to predict renal function loss after partial nephrectomy for cancer^18^. Paired T-test was used to compare kidney volumes before and after surgery. Bivariate and multivariable logistic regression analysis were used to assess prognostic factors influencing significant GFR loss (> 25%). Only significant factors in the bivariate analysis were included in the multivariable analysis. The analyses were performed using SPSS version 15.0 software (IBM Analytics, USA). 95% Confidence Interval (CI) and Odd-Ratio (OR) were used to report results. Alpha risk was 5%.

### Research involving human participants and/or animals

The clinical data reports for the study were collected and incorporated into the UroCCR database (French Research Network for Kidney Cancer, ClinicalTrials.gov Identifier NCT03293563), which is IRB-approved (*Comité Consultatif sur le Traitement de l'Information en Matière de Recherche dans le domaine de la Santé*) and obtained the CNIL (*Commission Nationale de l'Informatique et des Libertés*) authorization number DR-2013-206.

### Informed consent

Patients gave their informed consent.

## Results

### Patient and tumor characteristics

During this period, 137 patients were treated by LPNE. We excluded 44 patients (12 with single kidney and 28 without pre or post-operative CT-scan). Mean age was 60.4 years (SD 12.3), mean BMI was 27.4 kg/m^2^ (SD 5). According to R.E.N.A.L. tumor complexity score, 11 (11.8%), 46 (49.5%) and 36 (38.7%) tumors were respectively of high, moderate and low complexity. Mean tumor size was 3.4 cm (SD 1.6). In 4 cases (4.3%) the surgical margins were positive. The histology of the tumors is detailed in Table 1.

**Table 1:** Patient and tumor characteristics, peri-operative data.

Patient characteristics	
Mean age, year (SD)	60.4 (12.3)
Male, n (%)	57 (61.3)
Mean Body Mass Index, kg/m^2^ (SD)	27.4 (5)

Tumor characteristics	
**T stage, n (%)**	
T1a	63 (67.7)
T1b-2	30 (32.3)
**R.E.N.A.L. tumor complexity, n (%)**	
Low (1–6)	36 (38.7)
Intermediate (7–9)	46 (49.5)
High (10 and more)	11 (11.8)
Mean tumor size, cm (SD)	3.4 (1.6)
**Benign histology, n (%)**	16 (17.2)
Oncocytoma	10 (10.7)
Angiomyolipoma	4 (4.3)
Cystic	1 (1)
Metanephric adenoma	1 (1)
**Malignant histology, n (%)**	77 (82.8)
Clear cell renal cell carcinoma	51 (54.8)
Chromophobe renal cell carcinoma	8 (8.6)
Papillary renal cell carcinoma	17 (18.3)
Carcinoma of collecting duct	1 (1)
Positive surgical margins	4 (4.3)

### Peri-operative data

Mean total operative time was 153.0 min (SD 38). In 54 cases (58.1%) two or more subsegmental arteries (up to 4) were embolized. Mean volume of administrated iodinated contrast media was 68.5 mL (SD 68.5). Mean blood loss was 258.0 mL (SD 459), no hilar clamping was necessary. No secondary bleeding or embolization was reported. Perioperative data are reported on Table 1.

### Kidney function evolution

Pre-operative mean GFR-MDRD was 95.7 mL/min (SD 23.7), mean GFR-MDRD at 6 months was 86.6 mL/min (SD 22.9). Mean GFR-MDRD loss at 6 months after combined procedure was 9.4% (CI [6.7–12.5], *p* value < 0.001). Significant decrease of GFR-MDRD occurred in 10 patients (10.6%), 1 patient with no pre-operative kidney disease presented a new-onset chronic kidney disease. None of the patients required dialysis.

Mean pre-operative global, ipsilateral and contralateral renal volume were respectively 332.0 mL (SD 74.5), 164.0 mL (SD 40) and 168.0 mL (SD 39.2). Mean global, ipsilateral and contralateral renal volume at 6 months were respectively 315.6 mL (SD 72.4), 143.0 mL (SD 38.4) and 172.7 mL (SD 41.6). Global renal volume, ipsilateral and contralateral renal volume difference of means were significant, respectively − 16.5 mL (CI [10.2–22.7], *p* value < 0.001), − 21.0 mL (CI 16.8–25.3], *p* value < 0.001), + 4.7 mL (CI [− 7.9; − 1.5], *p* value = 0.004). Renal function and volume evolution are reported on Table 2.

**Table 2:** Renal function and volume evolution.

	Pre-operative	Follow-up 6 months
**GFR-MDRD**		
Mean GFR, mL/min (SD)	95.7 (23.7)	86.6 (22.9)
Difference of means, mL/min (SD) [t-test]		9.6 (15.1) [p < 0.001]
Significant renal function decrease, n (%)		10 (10.6)
Pre-operative chronic kidney disease, n (%)	4 (4.3%)	
New chronic kidney disease, n (%)		1 (1)
**Computed tomography renal volume**		
Mean global renal volume, mL (SD)	332 (74.5)	315.6 (72.4)
Difference of means, mL/min [t-test]		16.5 [p < 0.001]
Mean treated renal volume, mL (SD)	164 (40)	143 (38.4)
Difference of means, mL/min (SD) [t-test]		21 (20.6) [p < 0.001]
Mean contralateral renal volume, mL (SD)	168 (39.2)	172.7 (41.6)
Difference of means, mL/min (SD) [t-test]		4.7 (15.5) [p = 0.004]

Age was the only parameter significantly associated with > 25% loss of renal function (OR 1.075, CI [1.0–1.155], *p* value = 0.05). The mean operative time (OR 1.01, CI [0.99–1.03], *p* value = 0.32) and the mean parenchymal loss of operated kidney (OR 0.987, CI [0.95–1.02], *p* value = 0.435) were not correlated with significant loss of renal function. Bivariate and multivariable analysis of predictive factors of significant renal function decrease are respectively reported on Tables 3 and 4.

**Table 3:** Bivariate analysis of predictive factors of significant renal function decrease.

	< 25% RF loss	≥ 25% RF loss	p
Mean age, year (SD)	59.5 (12)	69.3 (7.8)	0.017
Mean BMI (SD)	27.1 (5)	29.7 (4.3)	0.138
BMI > 30, n (%)	28 (34)	5 (50)	0.318
**T stage, n (%)**			0.72
T1a	57 (69)	6 (60)	
T1b-2	26 (31)	4 (40)	
**R.E.N.A.L. tumor complexity, n (%)**			0.136
Low (1–6)	32 (38)	4 (40)	
Intermediate (7–9)	43 (52)	3 (30)	
High (10 and more)	8 (10)	3 (30)	
Mean tumor size, cm (SD)	3.3 (1.5)	3.7 (2)	0.56
Mean operative time, min (SD)	150.0 (37)	174.0 (39)	0.062
Mean Blood loss, mL (SD)	235.0 (451)	456.0 (497)	0.151
**Number of arteries embolized, n (%)**			0.86
1	35 (42)	4 (40)	
2 or more	48 (58)	6 (60)	
Mean iodinated contrast medium used, mL (SD)	68.4 (27)	68.8 (11)	0.97
Mean preoperative GFR, mL/min (SD)	95.7 (24)	95.4 (22)	0.96
Pre-operative Chronic kidney disease	3 (4)	1 (10)	0.371
Mean renal parenchyma loss, mL (SD)	19.0 (20)	31.0 (17)	0.077

**Table 4:** Multivariable analysis of predictive factors of significant renal function decrease.

	OR	CI	p
Mean age, year (SD)	1.075	1–1.155	0.05
Mean operative time, min (SD)	1.01	0.99–1.03	0.32
Mean renal parenchyma loss, mL (SD)	0.987	0.95–1.02	0.435

## Discussion

This descriptive study of functional renal outcomes after tumor vessels embolization and partial nephrectomy showed encouraging results. We found a moderate rate of renal function loss at 6 months estimated at 9.4%, considered to be in the high range but still comparable to known results after partial nephrectomy: a meta-analysis conducted in 2015 reported an average overall loss of renal function of about 10% after partial nephrectomy for patients with 2 kidneys^19^. We reported a moderate volume loss of the treated kidney at 6 months (about 12.5% i.e. 21.0 mL), these results are supported by Takagi et al. study (volume loss of 18%) and Mir et al. study (volume loss of 17%) after partial nephrectomy^20,21^. We reported minimal compensatory hypertrophy of the contralateral kidney of 2.9% (4.7 mL), also comparable to the work of Takagi et al. (2.2%) and Mir et al. (5%), appearing to be lower than in Jeon et al. (9.1%)^20–22^. As marked compensatory hypertrophy is a marker of parenchymal destruction of the operated kidney (contralateral hypertrophy of the order of 20% after radical nephrectomy), this minimal contralateral hypertrophy is an additional argument to confirm that embolization before partial nephrectomy is to be considered a nephron-sparing technique.

Whether or not to perform renal arterial clamping during partial nephrectomy is still debated, one meta-analysis seemed to show a better preservation of renal function in the short and medium term with the "off-clamp" technique, but another meta-analysis with a longer follow-up period showed that at 5 years the difference in renal function was no longer significant between the on-clamp and off-clamp methods^23,24^. However, if a clamp appears necessary to control bleeding, its duration (< 25 min) and selectivity will lead to better functional results^25^.

While arterial clamping remains controversial, preservation of the quantity and quality of renal parenchyma is essential^7,9^. This preservation first involves the surgical technique of tumor enucleation, which makes it possible to avoid removing healthy peritumoral parenchyma with acceptable oncological outcomes^26^. Second, this preservation is also the result of performing superselective embolization, supported by state-of-the-art tools such as CBCT, endovascular guidance software, microcatheters and embolization devices. The absence of any negative impact of the number of embolized arteries on post-operative renal function is a good illustration of this.

Although the margin of healthy embolized parenchyma is minimal, it is nevertheless sufficient to ensure satisfying haemorrhagic control, as evidenced by the mean blood loss we reported (258.0 mL), similar to that observed (276.8 mL) in a study performed with arterial clamping^27^. In this aforementioned study, despite the presence of arterial clamping, 16 patients out of 289 presented secondary bleeding requiring embolization, whereas no embolization for haemorrhage was reported in our work, confirming the results already published in a previous study^5,27^. The choice of glue as embolization material, filling the lumen of the distal arterioles, allows for immediate effective embolization and possibly more complete embolization than coils. These elements may explain the excellent control of bleeding shown in our work.

The multivariable analysis showed that only age is correlated with the occurrence of significant impairment of renal function, these results are corroborated by the results of Jeon et al. study^22^. Similarly, Lane et al. has shown that high age, among other non-modifiable factors, is associated with decreased post-operative renal function^9^. Elderly patients, regardless of the surgery performed, appear to have decreased renal function after surgery, probably related to a natural deterioration in renal function that would occur even if they did not undergo any surgical procedure.

Even though this procedure requires two operators working successively, the total operating time (153.0 min [SD 38]) is similar to conventional procedures (for example 141.3 min in George et al. work)^27^. This is possibly explained by the injection of blue dye into the tumor vessels, that facilitates tumor spotting through the peri-renal fat and the time saved due to the lack of renal hilum control^6^.

Considering the excellent control of haemorrhagic complications and observing the duration of hospitalisation, despite the cost of the material necessary for tumor vessels embolization, it would be interesting to compare the cost-effectiveness of our combined procedure versus a robot-assisted procedure.

Our study limitations include firstly the relatively short follow-up time after surgery. For our cohort it would be interesting to collect GFR-MDRD results 2 years after surgery to assess if a delayed recovery of renal function occurred as shown in Zabor et al. work which found that 2 years after surgery, 45% of patients had recovered renal function similar to pre-operative function, with a more likely recovery in female patients, having a large tumor size and an already impaired pre-operative renal function^28^. The second limitation is the small size of the cohort, a larger sample size could have allowed other lines of statistical analysis, such as the occurrence of new-onset chronic renal failure or a shift from moderate to severe chronic renal disease (GFR < 30 mL/min). Finally, we did not collect the presence or degree of albuminuria, which could have been of interest as a follow-up parameter or as a prognostic factor, as pointed out in the Huang et al. study^29^.

## Conclusion

The loss in GFR-MDRD and renal parenchyma volume (CTRV) after LPNE seems to be comparable to results already found in numerous published works in medical literature using other partial nephrectomy techniques. There was no correlation between the number of embolized arteries or renal parenchymal volume loss and renal function loss. Age was the only parameter significantly associated with > 25% loss of renal function. The main interest of this surgical technique is to facilitate the tumor excision while reducing the risk of bleeding.

## Data Availability

The clinical data reported for the study were collected within the framework of the UroCCR project (NCT03293563).
